# Notes on Dermatology

**Published:** 1894-12

**Authors:** Isadore Dyer

**Affiliations:** New Orleans, La., Professor of Dermatology in the New Orleans Polyclinic; Lecturer and Clinical Instructor in Skin Diseases, Medical Department Tulane University, Etc.


### Current Medical Literature.

### NOTES ON DERMATOLOGY.

BY ISADORE DYER, M. D., NEW ORLEANS, LA.,
Professor of Dermatology in the New Orleans Polyclinic; Lecturer and Clinical Instructor in Skin Diseases, Medical Department Tulane University, etc.

The *Medical Weekly* (Vol. II, No. 43) quoting from the *Monatsheft f. praktische Dermatologie*, of August 15, 1894, says that Dr. Bodara has found trichorrhexis on the increase among the women and young girls in Constantinople. The disease is distinguished: 1—By the development of minute greyish points at the ends of the hairs; 2—By the division of the hair into several filaments. At the affected point the hair becomes bent on itself, at an acute angle, and breaks on the slightest strain, or spontaneously. Bacteriological examination revealed the presence of a small rod-shaped bacillus, with slightly rounded ends, from 0.8 to 1.5″ in length and ⅓″ in width. The micro-organism was always surrounded by a clear zone, about ½′ in width, and a very delicately tinted membrane. Pure cultures are easily obtained and there is, therefore, no difficulty in studying the successive stages of development. In order to prepare a pure culture of the bacillus, the fragments of diseased hair should be steeped for five or six days in absolute alcohol. Then they should be placed on agar-agar, which becomes clouded in two or three days. The colonies appear in the form of round points, of a dirty gray or waxen color. The author proposes to call this micro-organism *bacillus multiformis trichorrhexeos.* Inoculation furnished positive and conclusive results. It would appear, then, that the trichorrhexis referred to, is a contagious parasitic disease, and differs in several particulars from the trichorrhexis nodosa of the text-books. Here there is fibrillary splitting only at the nodose points, while in Dr. Hadara's cases there was intermediary irregularity in the hair fibrillæ. Dr. Hodara is inclined to concede that the disease observed in Constantinople is distinct from the European or hitherto recognized variety.

---

Much of the November number of the *British Journal of Dermatology* is given to Dr. Leloir's article on "Dermato-Neuroses and their Treatment." He embraces in this class all cutaneous affections secondary to a modification either of the central, ganglionic or peripheral nervous system. The "purely sensory," the "purely motor," and the "vascular, or purely vaso-motor dermato-neuroses." In these classes are considered the hyperæsthetic and anæsthetic conditions, and such reflex disturbances as urticaria and the like.

Finally, the trophic and glandular dermato-neuroses, under the classes given by the author, include many conditions now included in the varieties of new growths, hypertrophies, atrophies, etc. While the article is only begun in this issue of the *Journal*, we must comment upon the strong determination of classes made by the author. Indirectly, certainly, many dermatoses are associated with a variety of sensory, motor, and even trophic disturbances; but it seems scarcely justifiable to determine the classification of these skin affections according to that nerve disturbance. The paper, however, must largely influence the arrangement of the general classes in the next attempt at a classification.

---

According to Dr. Leistikow (Hamburg) coal-tar possesses the advantage over wood-tar in the treatment of skin diseases. Its effect is more powerful and lasting, especially in pruriginous diseases. It is claimed to be an excellent means of treating dry eczemas of the scalp, neck, etc., and psoriasis, especially when the patches of the latter are situated on the scalp, knees or elbows. On the face the coal-tar is apt to produce an erythema, which would contra-indicate its use in persons who may have occupation demanding out-door life. The tincture of coal-tar recommended is as follows:

℞ Coal-tar ............................ ʒiss

Absolute alcohol .................... ʒi

Sulphuric ether ..................... ʒss

Mix. For external use.

When this tincture is applied with a brush to the affected parts, the alcohol and ether evaporate, leaving behind a fine layer of tar which can be rubbed off or removed with olive oil. (*Medical Weekly*, vol. ii, No. 44.)

---

Behrend (Berlin) considers that the difficulty in curing pityriasis versicolor is due in many instances to the popular impression that the disease is confined to the covered portions of the body, and that the disease on the exposed parts is a constant source of re-infection. Permanent lasting recovery, says Dr. Behrend, can only be realized by three agents, as far as is known, viz.: (1) tincture of iodine; (2) chrysarobin; pyrogallic acid. Tincture of iodine is objectionable on account of its liability to cause permanent pigmentation.

Pyrogallic acid is less effective than chrysarobin, and the author therefore gives preference to the latter. (*Ned. Wiek.*, Vol. ii, No. 44.)

All of this is very well, but to my mind chrysarobin and its stain, and likelihood to systemic intoxication is as objectionable as either the tincture of iodine or the pyrogallic acid. The author has completely ignored the factor clinically and fully demonstrated by Nielssen, of Copenhagen, last year. Nielssen demonstrated that the reinfection of pityriasis versicolor, apparently at regular intervals and seasons was strikingly coincident with the time at which the underclothing was changed, viz.: in the spring and fall. He elucidated from a number of cases that before the appearance of the eruption the patient had either worn newly bought underclothes, not having been previously washed, or else the underclothes worn had lain in a clothes press for a season. The fungus or micosporon furfur was found by him on such underclothing, dampness and absence of light favoring the development of the micro-organism. Simple parasiticides will destroy patches of pityriasis versicolor, and that in a short time. After reading Nielssen's article I began observations upon this class of cases and had the satisfaction of confirming his clinical evidence regarding the underclothes theory.

